# mRhubarb: Engineering of monomeric, red-shifted, and brighter variants of iRFP using structure-guided multi-site mutagenesis

**DOI:** 10.1038/s41598-019-52123-7

**Published:** 2019-10-30

**Authors:** Oliver C. Rogers, Dorothy M. Johnson, Elad Firnberg

**Affiliations:** Revolve Biotechnologies, Inc., Baltimore, MD USA

**Keywords:** Proteins, Imaging, Protein design, Molecular engineering

## Abstract

Far-red and near-infrared fluorescent proteins (FPs) enable *in vivo* tissue imaging with greater depth and clarity compared to FPs in the visible spectrum due to reduced light absorbance and scatter by tissues. However current tools are limited by low brightness, limited red-shifting, and a non-ideal dimeric oligomerization state. In this study we developed a monomeric variant of iRFP, termed mRhubarb713, and subsequently used a targeted and expansive multi-site mutagenesis approach to screen for variants with red-shifted spectral activity. Two monomeric variants were discovered, deemed mRhubarb719 and mRhubarb720, with red-shifted spectra and increased quantum yield compared to iRFP. These tools build on previously developed near-IR FPs and should enable improved *in vivo* imaging studies with a genetically encoded reporter.

## Introduction

Genetically encoded fluorescent proteins (FPs) have become vital life-science research tools. FPs based on green fluorescent protein (GFP) have been used for *in vivo* tissue imaging, cell tracing, and intracellular protein tracking. However, many of the GFP-based FPs suffer drawbacks including aggregation, photobleaching, and cytotoxicity which limits their utility for certain *in vivo* applications. Additionally, tissue autofluorescence and absorbance at the wavelengths at which these FPs operate (400–660 nm) creates high background and a low signal to noise ratio^1^. This limits their use to minimal surface cross-sections with a depth limit on single cell imaging of 0.05–0.1 mm and large tumor imaging up to 2.2 mm^2^. Brain imaging studies are constrained to 1–2 mm in imaging depth^3^. To overcome this limitation, neuroscientists must use highly invasive surgical methods to image deeper parts of the brain. More recently, FPs functional in the far-red and near-infrared (NIR) spectral region (635–720 nm) have been developed that can provide higher penetration depth with lower background tissue absorbance and autofluorescence. However, several issues remain to be addressed for an optimal NIR FP reagent, including improved brightness and increased red-shifting. Perhaps the most notable limitation with currently available FPs is their oligomeric state, as most FPs in the NIR range exist primarily in a dimeric conformation. This property hinders the usefulness of these tools as it restricts gene fusion partners to those that can tolerate the dimeric FP state without disruption of their natural function. In this study we aimed to improve upon these limitations by using a structure-guided combinatorial mutagenesis strategy to engineer a novel monomeric FP with increased brightness and red-shifted spectra.

Billiverdin IXα (BV)-based fluorescent proteins, such as those of the bacterial phytochrome protein (BphP) family, are useful templates for *in vivo* imaging applications. BphPs utilize BV, a linear tetrapyrrole bilin product of the oxidation of heme by heme oxygenase, as a chromophore. As such, BV is an abundant metabolic product in many species including insects, plants, and mammals and does not need to be provided exogenously. There is limited evidence that BV-based fluorescence has low cytotoxicity, despite concerns regarding perturbation of the endogenous BV pool. This was shown by previous studies comparing prolonged expression of iRFP variants to E2-Crimson (non-cytotoxic far-red FP control) and mKate2 (cytotoxic far-red GFP-like FP) in various mammalian cell lines. FACS analysis after 14- and 29-days post-transfection showed that cells expressing iRFP variants retained 55–84% of the initial fluorescence signal, similar to E2-Crimson, while 24% retention was observed for mKate2^4^. *In vivo*, iRFP fluorescence was detectable in mouse liver 10 days after infection by adenovirus containing the iRFP gene, suggesting that the FP is both stable and non-cytotoxic^5^. Furthermore, BV-based iRFP variants have higher photostability compared to most GFP-like FPs. Photobleaching half-time for five iRFP variants from iRFP670 to iRFP720 ranges from 290 to 960 s while EGFP, one of the most photostable GFP derivatives, is 174 s^4,6^. Finally, the NIR spectral properties of BV-based BphPs are highly advantageous for deep tissue imaging. Light absorption and scatter from hemoglobin, deoxyhemoglobin, water, and lipids reaches a minimum in the 700–900 nm NIR window^7^. Light at these wavelengths has greater incoming and outgoing tissue penetration depth compared to light in the visible spectrum. For these reasons, BV is a useful endogenous chromophore for *in vivo* imaging applications.

The BphP family of light-responsive proteins share common domain architecture consisting of a photosensory core domain (PCD) and a histidine kinase (HK) output effector domain involved in downstream signaling. The PCD is composed of three distinct domains, PAS (Per-Arnt-Sim), GAF (cGMP phosphodiesterase/adenylyl cyclase/FhlA) and PHY (phytochrome). PAS and GAF comprise the chromophore binding domain (CBD), which catalyzes the covalent attachment of BV to a PAS domain Cys residue via a thioester bond. The chromophore cycles between a red-absorbing Pr state with absorption in the 680–710 nm range and a far-red absorbing Pfr state in the 740–760 nm range. It should be noted that fluorescence in the Pfr state has not been observed. The Pr to Pfr photoconversion mechanism involves an isomerization process about the C15=C16 double bond between rings C and D of BV, changing its configuration from 15Z anti to 15E anti^7,8^. Additionally, a rotation of the entire chromophore alters the interactions with several residues in the CBD (Supplementary Table S1)^8^. Photoconversion dynamics suggests that conformational freedom allows the chromophore to dissipate its absorbed energy largely through rotational work rather than fluorescence emission. Prototypical phytochromes such as RpBph2 and DrBphP have a Pr ground state which photoconverts to Pfr upon illumination with red light. The bathy phytochromes, such as PaBphP, have a Pfr ground state which photoconverts to Pr upon illumination with far-red light. Return to the ground state occurs slowly via dark reversion or rapidly via illumination with far red or red light^8,10^.

For the template in this study we selected iRFP, a FP derived from the multi-domain bacterial phytochrome photoreceptor RpBphP2 from the purple non-sulfur bacteria *Rhodopseudomonas palustris*. iRFP is derived from the N-terminal 316 amino acids of RpBphP2 which retains the PAS and GAF domains while excluding PHY and HK. Selection for increased fluorescent brightness on a random mutagenesis library resulted in the identification of 13 substitutions in the original template^5^. Due to these mutations and its lack of the PHY domain, iRFP is locked in the Pr state and does not photoconvert. iRFP has an excitation maximum of 690 nm and an emission maximum of 713 nm with a quantum yield of 6.3% and photostability of 960 s. A further engineered variant, iRFP720, with excitation/emission of 702/720 nm had four additional mutations with slightly lower quantum yield (6.0%) and photostability (490 s)^4^. Other variants, such as mIFP, miRFP703, miRFP709 and miRFP720 have been developed which have been shown to be monomeric in nature. However, these proteins suffer decreases in molecular brightness compared to iRFP^9^.

In order to create a monomeric, red-shifted variant of iRFP without sacrificing brightness, we used a high-throughput combinatorial library screening approach using PFunkel multi-site mutagenesis. We selected the PFunkel technique as it enables rapid construction of large user-defined combinatorial libraries in plasmid format. We used information from BphP-family crystal structures, mutagenesis studies, and *in silico* models to guide the selection of target residues that could modulate BV conformation and bias it towards a 15E Pfr fluorescent state.

## Results

**Figure 1:**
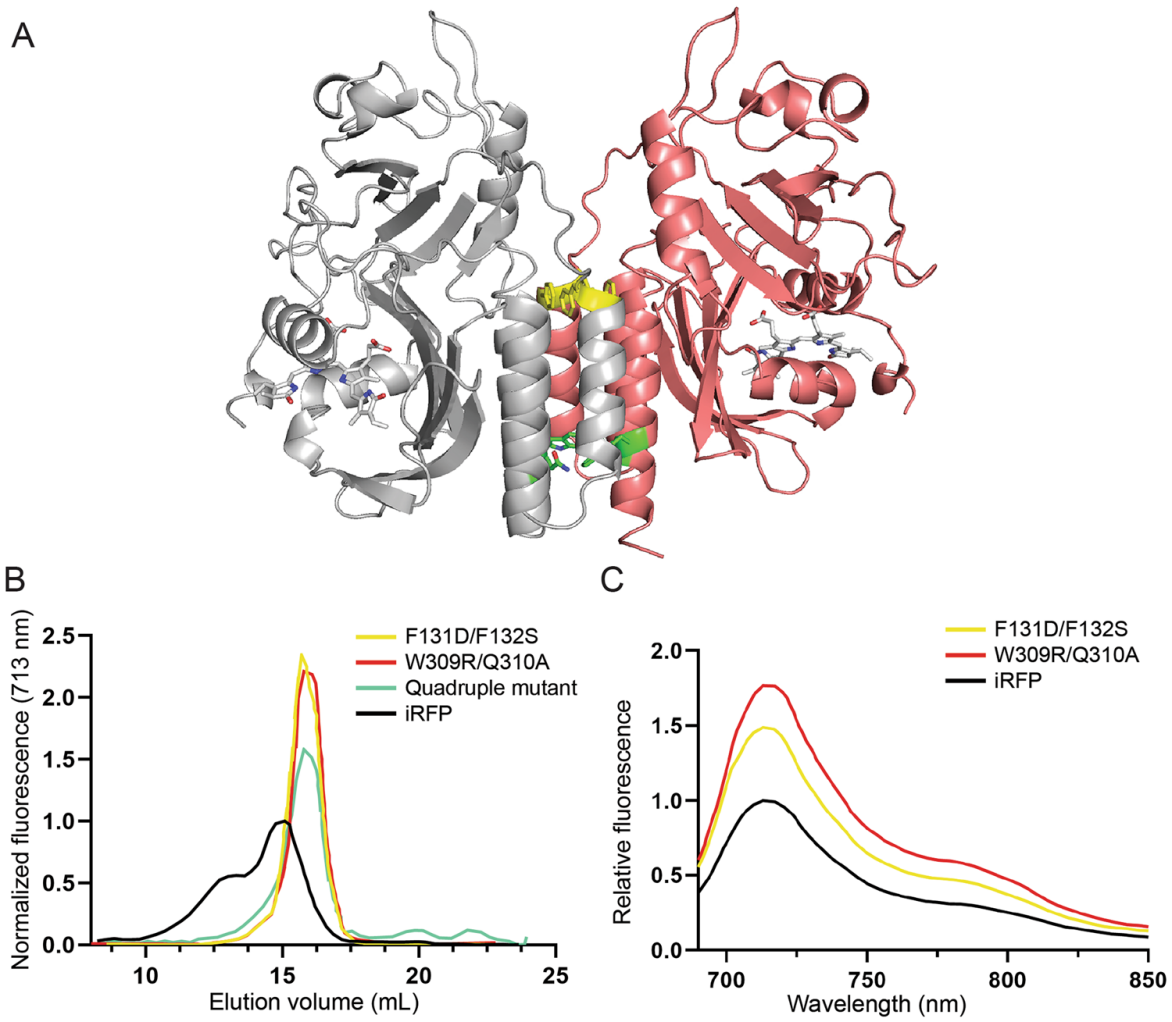
(**A**) Homology model of iRFP based on the crystal structure of the PAS and GAF domain from RpBphP2 (4E04.pdb). Mutations that disrupt the dimer interface are shown in yellow (F131S/F132D) and green (W309R/Q310A). The BV chromophore is shown in the binding pocket covalently attached to the PAS-domain Cys and cradled by the GAF domain within each identical monomer, shown in gray and pink. The putative dimerization interface is comprised of three helices forming a helix bundle that buries a significant surface comprised of both hydrophobic and charged residues. (**B**) Gel filtration elution profile with curves normalized to area under the curve to eliminate any differences in amount of loaded protein. Fluorescence measurements were taken using excitation at 690 nm and emission at 713 nm. (**C**) Fluorescence emission scans for iRFP, F131S/F132D and W309R/Q310A mutants.

Given its ability to fluoresce in the NIR window *in vivo*, iRFP is a useful template from which to design new tools to monitor a variety of cellular processes. iRFP is reported to be a dimer in solution based on the crystal structure of RpBphP2^4^. To engineer this protein into a purely monomeric state, we used homology-guided modelling to identify key residues involved in the dimerization process. An atomic resolution homology model of iRFP bound to BV in the red-absorbing Pr state was created using Modeller software based on the crystal structure of the chromophore-binding domain of RpBphP2 (PDB# 4E04), with which iRFP shares 91% identity (Fig. 1A). This model was used to guide a point mutation strategy to create an iRFP monomer. Analysis of the homodimer buried interface shows two pairs of residues F131/F132 and W309/Q310 that contribute significant interactions to the interface. The F131 and F132 residues pi-stack with their adjacent counterparts. Near the C-terminus, W309 and Q310 sit at the bottom of the interface such that the Trp residues stack.

We assessed the oligomerization state of iRFP via gel filtration chromatography and found that the protein elutes as two distinct peaks, suggesting iRFP exists as a heterogeneous mixture of two separate oligomerization states under conditions tested (Fig. 1B). This heterogeneity can complicate the interpretation of results from efforts to engineer novel activities into iRFP and disrupt structure-function relationships of a protein of interest when expressed as a fusion. To disrupt dimerization, we then used site-directed mutagenesis at the aforementioned interface residue positions. In one mutant, F131S and F132D mutations were introduced to remove the hydrophobicity and bulkiness of the segment while creating easily solvated charged interactions that would repulse the adjacent monomer. In a second mutant, W309R and Q310A mutations were introduced to create steric clash and abolish the charged interaction across the dimerization helices. Analysis of gel filtration elution curves demonstrated the F131S/F132D and W309R/Q310A mutants retain fluorescence and each elute as a single monomeric peak that is shifted towards a smaller apparent mass relative to the wild-type iRFP (Fig. 1B). Thus, these changes in the putative dimerization interface produced the effect of shifting the oligomerization state from a mixed population to a monomeric state.

Surprisingly, both W309R/Q310A and F131S/F132D mutants produced monomers with increased brightness compared to wild-type iRFP as determined by fluorescence spectral scans (Fig. 1C). The monomeric W309R/Q310A mutant, henceforth name mRhubarb713, has a quantum yield of 7.63% and relative molecular brightness of 140.1% compared to iRFP (Table 1). The W309R/Q310A/F131S/F132D quadruple mutant produced a monomer that was not brighter than wild-type iRFP (data not shown). Typically, oligomeric and dimeric proteins are brighter than their monomeric counterparts due to more efficient folding and incorporation of the chromophore. For example, the red FP derived from *Discoma* coral DsRed was initially isolated as obligate oligomers, lost almost all fluorescence upon monomerization, and required extensive engineering to brighten^10^. In the case of iRFP, we hypothesize that interactions between secondary structural elements of the CBD and the chromophore may be enhanced by monomerization resulting in decreased BV conformational freedom and increased fluorescence. Indeed, the native RpBphP2 functions by converting light induced conformational changes in BV into energy to modulate the function of PHY and HK domains^11^. The dimer may be important for facilitating this light induced work.

**Table 1:** Spectral properties of iRFP variants ND denotes parameters that could not be determined due to lack of fluorescence or fluorescent signal below the limit of quantitation.

Variant	Mutations	Oligomeric state	Excitation maximum (nm)	Emission maximum (nm)	Extinction Coefficient (M^−1^ cm^−1^)	Stoke’s shift (nm)	Quantum yield (%)	Quantum yield relative to iRFP	Molecular brightness vs iRFP (%)	Brightness vs. iRFP in BL21 *E. coli* (%)
iRFP (iRFP713)	—	Dimer	690	713	98,000	23	6.30	1	100	100
iRFP720	E180S, V203I, V254N	Dimer	702	720	96,000	18	6.00	0.95	93.3	Not tested
miRFP720	E180S, V203I, E301K, L302R, Q305E, V306R, W309T	Monomer	702	720	98,000	18	6.10	0.97	96.8	Not tested
IFP2.0	DrBphP Template	Mixed	691	711	86,125	20	8.00	1.27	111.6	Not tested
mRhubarb713	R134H, W309R, Q310A	Monomer	690	713	113,457	22	7.63	1.21	140.1	85.6
mRhubarb719	R134H, Y198S, T202Y, V203I, W309R, Q310A	Monomer	700	719	83,768	19	6.80	1.08	92.2	49.4
mRhubarb720	R134H, L196Q, T202D, V203I, W309R, Q310A	Monomer	701	720	94,941	19	6.46	1.03	99.3	68.5
1A7	R134H, T202D, V203I, T267A, W309R, Q310A	Monomer	693	717	85,544	14	5.57	0.88	77.2	35.0
1A9	R134H, T202D, V203I, F258Y, T267A, I282F, W309R, Q310A	Monomer	693	717	14,717	24	2.60	0.41	6.2	9.9
2D7	R134H, L196Q, T202D, V203I, V283S, W309R, Q310A	Monomer	705	720	96,971	15	3.07	0.49	48.3	25.0
3A2	L196Q, T202D, V203I, F258Y, V283S, W309R, Q310A	Monomer	692	713	87,089	21	6.10	0.97	86.0	15.6
3D5	L196Q, T202D, V203I, F258Y, W309R, Q310A	Monomer	697	715	31,004	18	2.50	0.40	12.6	62.2
3C5	R134H, L196Q, T202D, V203I, F258Y, T267A, I282F, V283S, W309R, Q310A	Monomer	ND	ND	ND	ND	ND	ND	ND	ND
2F8	R134H, Y171A, L196Q, T202D, V203I, S269A, W309R, Q310A	Monomer	ND	ND	ND	ND	ND	ND	ND	ND
3A4	R134H, L196Q, T202D, V203I, T267S, I282F, V283S, W309R, Q310A	Monomer	703	720	36,012	17	4.8	0.76	28.0	4.90
3A4-F258Y	R134H, L196Q, T202D, V203I, F258Y, T267S, I282F, V283S, W309R, Q310A	Monomer	ND	ND	ND	ND	ND	ND	ND	2.70
3A4-S269A	R134H, L196Q, T202D, V203I, T267S, S269A, I282F, V283S, W309R, Q310A	Monomer	ND	ND	ND	ND	ND	ND	ND	ND

**Figure 2:**
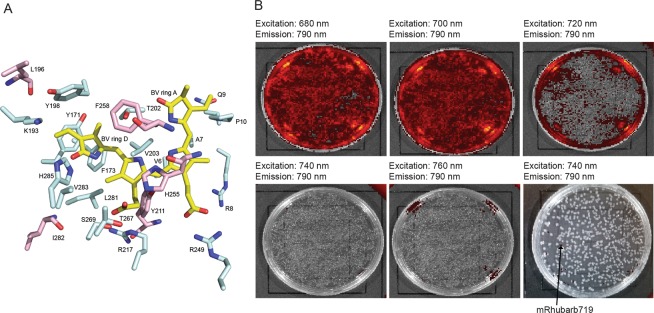
(**A**) Homology model of iRFP showing the BV chromophore (yellow) and residues mutated in set 1 libraries (blue) and additional residues mutated in set 2 libraries (pink). (**B**) IVIS images of cells transformed with library 1 imaged with increasing excitation wavelengths (top row and bottom two left). IVIS image of the plate in which mRhubarb719 was identified is shown bottom right.

**Figure 3:**
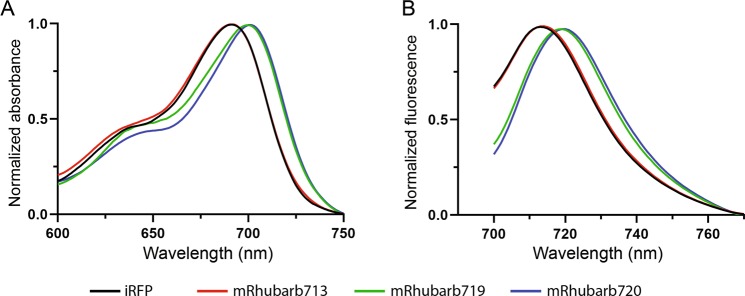
(**A**) Absorbance spectra and (**B**) fluorescence emission spectra for iRFP, mRhubarb713, mRhubarb719, and mRhubarb720 using an excitation wavelength of 400 nm (the Soret band). All values have been normalized to the maximum value for each protein.

A second homology model representing iRFP bound to BV in the far-red-absorbing Pfr state was generated based on the crystal structure of PaBphP (PDB# 3C2W) in the Pfr state. Both model structures were energy minimized and aligned for comparison. Several interactions between iRFP amino acid side chains in the CBD differed between the Pr and Pfr models. These sites provided a starting point for saturation mutagenesis in order to shift conformation towards the Pfr state. Based on these models, the sites chosen for randomization were V6, A7, R8, Q9, P10, Y171, F173, K193, Y198, T202, V203, R217, R249, T267, S269, L281, V283, and H285 (Fig. 2A, residues for set 1 libraries). The NNC codon was chosen for randomization at each site, which provides access to 15 amino acids while avoiding stop codons. Fifteen libraries were generated using PFunkel mutagenesis which introduced randomization in different combinations of the above positions. Efficiency of PFunkel reactions was assessed by running each step of the reaction on an agarose gel (Supplementary Fig. S1). Library transformation yields ranged from 10^4^ to 10^6^ colonies and a total of approximately 1–2 million colonies were screened. Following plating on LB-agar bioassay dishes and NIR plate imaging, fluorescent colonies suspected of red-shifting were picked, sequenced, and expressed for further analysis. The main challenge we encountered with this screening approach is the difficulty in distinguishing colonies with enhanced brightness or expression (of which there were many) from colonies with truly red-shifted emission spectra (of which there were few). Nevertheless, a mutant identified from this screen (shown in Fig. 2B) harbored three mutations, Y198S, T202Y, V203I with an excitation peak of 700 nm, emission peak of 719 nm, quantum yield of 6.80% and relative molecular brightness of 92% compared to iRFP, and was termed mRhubarb719 (Fig. 3, Table 1).

Due to the signal-to-noise challenges encountered when screening large libraries on plates, a focused combinatorial mutagenesis approach was developed based on homologous residues previously shown to play critical roles in forming the PaBphP Pfr and Pr states (Supplementary Table S2). PaBphP has a Pfr ground-state absorbance peak of 751 nm and has been crystallized in both dark Pfr and light Pr states^8,12,13^. Mutagenesis studies have shown which residues play critical roles for stabilizing and destabilizing the Pfr state (Supplementary Information). Thus, the rationale for this strategy was to bias the chromophore conformation towards a fluorescent Pfr state by introducing mutations that stabilize the 15E conformation while destabilizing 15Z (Fig. 2A, residues for set 2 libraries). PFunkel multi-site mutagenesis was used to make a focused library of all 4096 possible combinations of 12 mutations important for photoconversion dynamics. Individual clones were grown in 96-well format and screened by spectral analysis in a Spectramax fluorescence platereader. Mutants displaying red-shifted emission spectra were selected, sequenced and expressed for further analysis. This screen identified 9 mutants with red-shifted spectral characteristics as shown in Table 1, one of which, deemed mRhubarb720 (additional mutations L196Q, T202D, V203I) had an excitation peak of 701 nm and an emission peak of 720 nm (Fig. 3). This variant also had favorable quantum yield of 6.46% and brightness of 99% compared to iRFP making it the brightest, red-shifting mutant found in this study (Table 1).

Mutations T202D and V203I, which are reversions to the original RpBph2 sequence, were found in all red-shifted variants tested including mRhubarb720 (Table 1). However, most of these had reduced brightness. While D202 is noted as a critical residue for forming the Pfr state, high intensity Pr-based fluorescence was only noted in PaBphP once the homologous D194A mutation was introduced^13^. Thus, the reduced brightness upon mutational reversion is consistent with previous findings. The mutations present in non-fluorescent clones from the library were also analyzed. It was observed that Y171A and R249A abolished fluorescence, indicating that these residues are critical for activity (data not shown).

The arm of the PHY domain is known to be critically involved in forming the Pfr state. Specifically R453 and S459 in PaBphP (R462 and S468 in RpBphP2) stabilize the flipping of the BV ring D. Mutations R453A and S459A have been showed to disrupt formation of the Pfr state^13^. Therefore, we hypothesized that reintroducing this portion of the PHY domain into iRFP could stabilize the Pfr state without inducing the signaling conformational change of the full-length phytochrome. We generated multiple red-shifted mutants in which the PHY arm from PaBphP E431-S471 was fused to the N-terminus with a GGGGS linker and to the C-terminus with a (GGGGS)x2G linker. However, when expressed in *E. coli* under all conditions tested, these mutants’ fluorescence was below the limit of quantitation.

To assess the properties of each mutant in bacterial cells, we expressed these constructs in BL21 *E. coli* and compared their brightness in cells relative to iRFP. The iRFP template yielded the brightest transformed *E. coli* when compared to the other mutants including mRhubarb713, which had notably improved molecular brightness *in vitro* (Table 1, Supplementary Fig. S2). The reason for this discrepancy is unclear, however, it may suggest that dimeric structure of iRFP promotes stability and improved expression in cells. The fluorescent properties of each mutant will vary in different cell and tissue types. Therefore, the optimal variant should be selected and expression conditions optimized for each specific cell model. Future *in vivo* studies are warranted to compare these novel variants to previous FPs.

## Discussion

The need for improved imaging reagents that allow for deeper tissue imaging at higher resolutions has led to a variety of fluorescent proteins with increasingly red-shifted spectra. In this study, we developed three monomeric variants of iRFP, termed mRhubarb713, mRhubarb719, and mRhubarb720 all of which had improved brightness or red-shifting compared to iRFP. This was enabled by two expansive multi-site combinatorial mutagenesis screens guided by structural information of the protein. Of these two methods, the more randomized (set 1), high-throughput approach generated hundreds of thousands of iRFP mutants. Unfortunately, due to a high false-positive rate, this approach was less fruitful and only one useful mutant, mRhubarb719 was discovered. A narrower mutagenesis strategy (set 2) focused on stabilizing the Pfr state of the protein enabled discovery of multiple red-shifted variants of iRFP. Similar future studies should base their screening methods on the latter strategy until a more user-friendly imaging assay can be developed.

This work demonstrates the ability to both red-shift emission spectra and increase quantum yield using structure-guided engineering. Future efforts will focus on further red-shifting to achieve a bright emission maximum near 800 nm. This wavelength represents a “sweet-spot” where tissue absorbance and autofluorescence is at a minimum. While it may be possible to further red-shift Pr-based fluorescence, larger red-shifts may come from utilizing the far-red absorbing Pfr state. It is likely that the red-shifted mutants discovered in this study still reside in the 15Z conformation of the Pr state. This is evidenced by the observation that the R249A mutation abolishes fluorescence in various mutational backgrounds. R249 is known to hydrogen bond with the ring B propionate group of BV in the Pr state^14^. Fluorescence in the Pfr state has not yet been demonstrated, thus suggesting that the chromophore in native BphPs dissipates its absorbed energy through rotational work rather than fluorescence emission. Mutations that promote Pr-based fluorescence fix this conformation state and prevent photoconversion to Pfr^11^. Thus, if the appropriate mutations or modifications of the CBD can fix the chromophore in the 15E anti conformation, Pfr-based fluorescence may yet be achieved.

## Methods

### Constructs

The gene for iRFP was synthesized (Integrated DNA Technologies, Coralville, IA) and cloned into the pETDUET-1 vector (Novagen, Madison, WI) co-expressing heme oxygenase (for generation of the BV chromophore) from two separate T7/lacO promoters. The N-terminal residues of the iRFP gene are MGSS-H_6_-SQDP which includes the 6xHis tag and excess residues from the multiple cloning site. Additionally, the iRFP gene contains a point mutation resulting in an R134H mutation which did not affect activity. W309R/Q310A and F131S/F132D mutants were created using the QuickChange protocol (Agilent Technologies, Santa Clara, CA).

### Expression and purification of iRFP mutants

For gel filtration, iRFP variants were expressed in *E. coli* BL21(DE3) cells. A freshly transformed colony was picked and grown in 10 mL LB with carbenicillin 100 μg/mL overnight at 37 °C with shaking, then 1/100^th^ volume used to inoculate LB media containing carbenicillin 100 μg/mL. The culture was incubated at 37 °C shaking at 250 RPM until an OD600 of 0.4 to 0.8 was reached. Protein expression was induced by addition of 500 µM IPTG and incubation at 22 °C shaking at 250 RPM for 16 hrs. The cells were centrifuged at 4000 × g for 20 minutes and lysed by resuspending the cell pellet in 1 mL BugBuster Master Mix protein extraction reagent (Novagen, Madison, WI) containing SIGMAFAST protease inhibitor cocktail (S8830, Sigma-Aldrich, Saint Louis, MO). The mixture was incubated at room temperature for 30 minutes and centrifuged at 18,000 × g for 30 minutes to remove insoluble material. The clarified lysate was then mixed with Hispure Ni-NTA resin (88221, Thermo Scientific) for 30 minutes, loaded onto a 5 mL disposable column, washed with PBS pH 7.4 containing 20 mM imidazole, and eluted in PBS containing 250 mM imidazole. Total protein concentration was determined using the Bradford assay. Concentration of all mutants was determined by band densitometry analysis using ImageJ software (Supplementary Fig. S3). All protein preps were run on SDS PAGE using Mini-PROTEAN TGX pre-stained gels (4568084, Bio-Rad, Hercules, CA). Glycerol stocks of iRFP and all mutants were stored at −80 °C.

### Gel filtration

Ni-NTA purified iRFP mutants were further purified using a Superdex 200 26/60 gel filtration column (Amersham Biosciences, Little Chalfont, United Kingdom) run on a GE ÄKTA FPLC equilibrated in 50 mM Tris-Cl (pH 8.0), 500 mM NaCl at 4 °C. The elution profile of iRFP mutants was characterized by collecting elution fractions followed by fluorescence measurement on Biotek HM fluorometer using excitation/emission wavelengths of 690/713 nm. Peak fractions were collected and dialyzed against 5 mM Tris-Cl (pH 8.0) and 100 mM NaCl, concentrated to 10 mg/ml using an Amicon Centricon 10 column (Millipore, Billerica, Massachusetts, United States). Data was normalized to area under the curve for each sample and scaled such that the maximum of the curve for iRFP was 1.0.

### *In silico* modelling of iRFP

Since a crystal structure of iRFP is not available, homology modeling of iRFP was performed using MODELLER software based on the RpBphP2 CBD in the Pr state (PDB# 4E04)^12,13^. A second homology model representing iRFP bound to BV in the Pfr state was generated based on the PaBphP CBD in the Pfr state (PDB# 3C2W). Both model structures were energy minimized, validated and aligned for comparison. All figures were created using PyMol.

#### PFunkel mutagenesis

pET-DUET-1-iRFP-HO plasmid ssDNA was generated as previously described^4,15^. PFunkel multi-site mutagenesis was performed as previously described with minor modification^5,16^. Briefly, a 100 μL reaction was prepared in a 0.5 mL microtube containing 1X PfuTurbo Cx Hotstart DNA polymerase buffer, 5% DMSO, 0.2 mM dNTPs, 0.5 mM NAD+ , 1 mM MgCl_2_, 140 nM phosphorylated oligos, and 4.6 nM ssDNA template. The reaction was heated to 95 °C for 2 min, 63.6 °C for 10 min. Then 0.025 U/uL PfuTurbo Cx Hotstart DNA polymerase and 2 U/uL Taq ligase, pre-activated by heating to 95 °C were added to the reaction. Temperature cycling proceeded at 63.6 °C for 60 min, 45 °C for 15 min, 4 °C for 10 min, 95 °C for 2 min, 45 °C for 15 min, 4 °C hold. Then 0.10 U/uL uracil DNA glycosylase, 0.29 U/uL exonuclease III, and 0.38 U/uL exonuclease I were added to the reaction and incubation continued at 37 °C for 60 min. The reaction was heated to 95 C for 2 min, during which 50 nM secondary phosphorylated oligo was added followed by incubation at 63.6 °C 30 min, and 45 °C 15 min. Aliquots were withdrawn after step 1 (extension/ligation), step 2 (template strand degradation), and step 3 (secondary extension/ligation) to validate correct product formation on an agarose gel (Supplementary Fig. S1). The product DNA was purified using the Nucleospin Gel and PCR clean-up kit (Macherey-Nagel, Düren, Germany). E. cloni EXPRESS BL21(DE3) cells (Lucigen, Middleton, WI) cells were transformed with product DNA, plated on agar plates containing ampicillin 100 μg/mL and grown at 37 °C overnight.

#### On-plate screen

Fifteen combinatorial libraries were generated using PFunkel mutagenesis utilizing different combinations of mutagenic primers at the residues V6, A7, R8, Q9, P10, Y171, F173, K193, Y198, T202, V203, R217, R249, T267, S269, L281, V283, and H285 (primers in Supplementary Table S2). Libraries were plated on LB-agar with 50 μM IPTG on bioassay dishes at a density of 10^4^–10^6^ CFU/plate via transformation of high efficiency E. cloni T7 EXPRESS BL21 (DE3) electrocompetent cells (Lucigen Corporation, Middleton, WI) according to manufacturer’s instructions. After overnight colony growth and protein expression, plates were imaged on the IVIS Lumina III instrument using an excitation wavelength of 680, 700, 720, 740, and 760 nm with an emission wavelength of 790 nm. All colonies were compared to the brightness of the wildtype iRFP colonies. Colonies were picked and grown in LB containing ampicillin for further analysis.

#### 96-well screen

PFunkel multi-site mutagenesis was used to make a focused library making all possible combinations of Y171A, L196Q, F198Y, T202D, V203I, Y211A, R249A, H255A, F258Y, T267A, S269A, I282F, and A283S (primers in Supplementary Table S2). Individual colonies were seeded into 1 mL of 2xYT in 96 deep-well plates and grown overnight at 37 °C with shaking. Ten microliters of culture were transferred to 1 mL of 2xYT in a 96 deep-well plate and incubated at 37 °C with shaking at 250 rpm until reaching an OD of 0.4–0.8. Expression was induced by addition of IPTG to 500 μM and incubation continued at 22 °C overnight with shaking at 250 rpm. Cultures were pelleted at 4500xg for 10 min and the supernatant withdrawn. The pellets were resuspended in 200 μL PBS and transferred to a 96 black-well plate. Emission spectra in culture format were read on a Spectramax M2 platereader (Molecular Devices, Sunnyvale, CA) with excitation at 400 or 650 nm with a 695 nm cutoff and emission scan from 700–800 nm.

### Fluorescence characterization assays

To characterize each mutant, purified variants were diluted in PBS with 250 mM imidazole to a concentration of 0.6 mg/mL. Two-fold serial dilutions of the solution were made to generate a concentration gradient. Samples were scanned to find their emission maxima using an excitation wavelength of 400 nm and emission wavelengths of 670–770 nm. To determine the excitation maximum for each mutant, absorbance scans were taken at wavelengths ranging from 600–750 nm. Quantum yield was determined by measuring the absorbance and total integrated fluorescence (area under the curve) for each mutant at four protein concentrations. These points were then plotted and the slope of the linear regression calculated. Quantum yield was calculated by multiplying the ratio in slopes between the mutant and iRFP by iRFP’s quantum yield^18^. The brightness relative to iRFP was then calculated as the product of the quantum yield and the extinction coefficient.

### Fluorescence in *E. coli*

BL21 DE3 *E. coli* were transformed with plasmids for all mutants displaying improved brightness or red-shifting compared to iRFP. Single colonies were picked and inoculated overnight at 37 °C in 5 mL terrific broth medium shaking at 250 RPM. No IPTG was added, since leaky expression alone was found to provide sufficient fluorescence under these growth conditions. Cells were centrifuged at 5000 × g for 10 minutes and resuspended in 0.2 mL PBS. Emission spectra were then collected using an excitation wavelength of 400 nm. Total brightness in cells was then corrected to the number of cells present in the culture via the OD600 of each culture diluted one thousand-fold (or until OD600 = 0.5–1.0).

## Supplementary information

Supplementary Information

## Data Availability

The datasets generated and/or analyzed during the current study are available from the corresponding author upon reasonable request.

## References

[1] Day RN, Davidson MW (2009). The fluorescent protein palette: tools for cellular imaging. Chem Soc Rev.

[2] Yang M (2000). Whole-body optical imaging of green fluorescent protein-expressing tumors and metastases. Proc. Natl. Acad. Sci. USA.

[3] Kim G (2017). Deep-brain imaging via epi-fluorescence Computational Cannula Microscopy. Sci Rep.

[4] Shcherbakova DM, Verkhusha VV (2013). Near-infrared fluorescent proteins for multicolor *in vivo* imaging. Nat Meth.

[5] Filonov GS (2011). Bright and stable near-infrared fluorescent protein for *in vivo* imaging. Nat Biotech.

[6] Shaner NC, Steinbach PA, Tsien RY (2005). A guide to choosing fluorescent proteins. Nat Meth.

[7] Khullar O, Frangioni JV, Grinstaff M, Colson YL (2009). Image-guided sentinel lymph node mapping and nanotechnology-based nodal treatment in lung cancer using invisible near-infrared fluorescent light. Semin. Thorac. Cardiovasc. Surg..

[8] Yang X, Kuk J, Moffat K (2009). Conformational differences between the Pfr and Pr states in Pseudomonas aeruginosa bacteriophytochrome. Proc. Natl. Acad. Sci. USA.

[9] Shcherbakova DM, Cammer NC, Huisman TM, Verkhusha VV, Hodgson L (2018). Direct multiplex imaging and optogenetics of Rho GTPases enabled by near-infrared FRET. Nature Chemical Biology 2018 14:6.

[10] Campbell RE (2002). A monomeric red fluorescent protein. Proceedings of the National Academy of Sciences.

[11] Samma AA, Johnson CK, Song S, Alvarez S, Zimmer M (2010). On the origin of fluorescence in bacteriophytochrome infrared fluorescent proteins. J Phys Chem B.

[12] Rockwell NC, Shang L, Martin SS, Lagarias JC (2009). Distinct classes of red/far-red photochemistry within the phytochrome superfamily. Proceedings of the National Academy of Sciences.

[13] Yang X, Kuk J, Moffat K (2008). Crystal structure of Pseudomonas aeruginosa bacteriophytochrome: Photoconversion and signal transduction. Proceedings of the National Academy of Sciences.

[14] Bellini D, Papiz MZ (2012). Dimerization properties of the RpBphP2 chromophore-binding domain crystallized by homologue-directed mutagenesis. Acta Crystallogr. D Biol. Crystallogr..

[15] Tonikian R, Zhang Y, Boone C, Sidhu SS (2007). Identifying specificity profiles for peptide recognition modules from phage-displayed peptide libraries. Nature Protocols.

[16] Firnberg E, Ostermeier M (2012). PFunkel: efficient, expansive, user-defined mutagenesis. PLoS ONE.

