# Supplementary material for: mRhubarb: Engineering of monomeric, red-shifted, and brighter variants of iRFP using structure-guided multi-site mutagenesis
Source: Sci Rep. 2019 Oct 30;9:15653. doi: 10.1038/s41598-019-52123-7 (PMC6821797; doi:10.1038/s41598-019-52123-7)
Supplement: Supplementary file 1 — Supplementary Information [file 41598_2019_52123_MOESM1_ESM.pdf]

mRhubarb: Engineering of monomeric, red-shifted, and brighter variants of iRFP using structure-guided multi-site mutagenesis

Oliver C. Rogers, Dorothy M. Johnson, Elad Firnberg

Supplementary Information

Supplementary information

The bathy phytochrome PaBphP from *Pseudomonas aeruginosa* has a Pfr ground state which photoconverts to Pr under far-red light illumination. Crystal structures of PaBphP show the Pfr dark state is stabilized through hydrogen bond interactions between the pyrrole nitrogen of ring D with D194 and Y250 and between the carbonyl group of ring D with Y250, S459, and Q188. D194A and H277A mutations trap the phytochrome in a Pr dark state with no Pfr photoconversion, while Y250F, Q188L, and S275A maintain the Pfr dark state but increase the reversion time from Pr back to Pfr. S261A traps the Pfr dark state and prevents photoconversion to Pr, while Y163A and R241A reduce reversion time back to Pfr. S459A and R453A mutants form a dark Pr state with very limited photoconversion to Pfr. Additionally, R453 plays a role by stabilizing D194 through a hydrogen bonding interaction. Both R453 and S459 are located in an arm of the PHY domain and are critical for forming the Pfr state[9,14]. Crystal structure of the photoactivated Pfr state of prototypical phytochrome DrBphP from *Deinoccocus radiodurans* shows that homologous residues D207, S468 and Y263 (D194, S459, Y250 respectively in PaBphP) stabilize the 15E chromophore conformation but do not form direct interactions with the chromophore as in PaBphP[17].

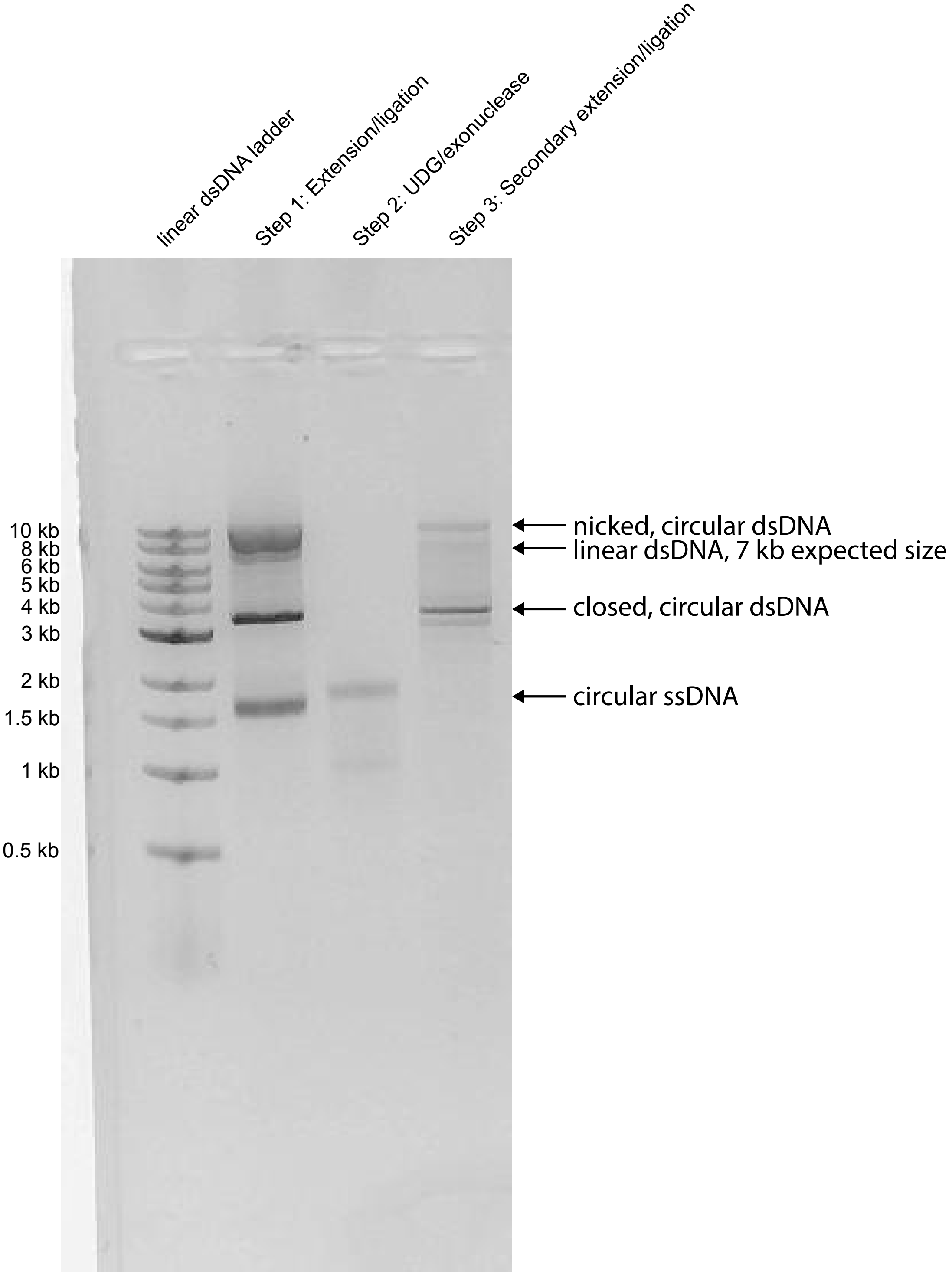

Supplementary Figure S1. Agarose (0.8%) DNA gel showing the steps of a PFunkel mutagenesis library reaction. In step 1, template plasmid dU-ssDNA is incubated with mutagenic primers and polymerase/ligase to generate a double-stranded hetero-duplex. In step 2, the wildtype template strand is degraded using a UDG/exonuclease cocktail leaving the mutated ssDNA. In step 3, a secondary primer is used to generate the final dsDNA PFunkel library product.

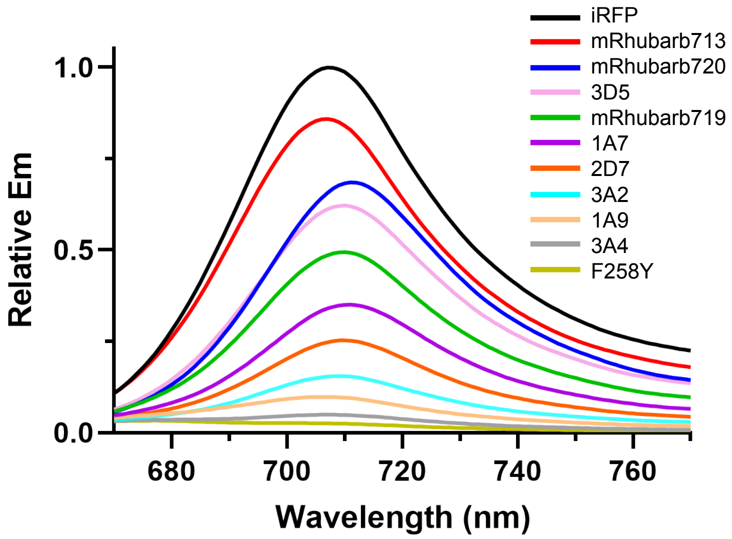

Supplementary Figure S2: Emission spectra for all red-shifted or brighter mutants of iRFP in BL21 *E. coli*. Fluorescence was corrected to the number of cells present based on the relative OD600 of iRFP cultures diluted 1000-fold. All samples were excited at a wavelength of 400 nm.

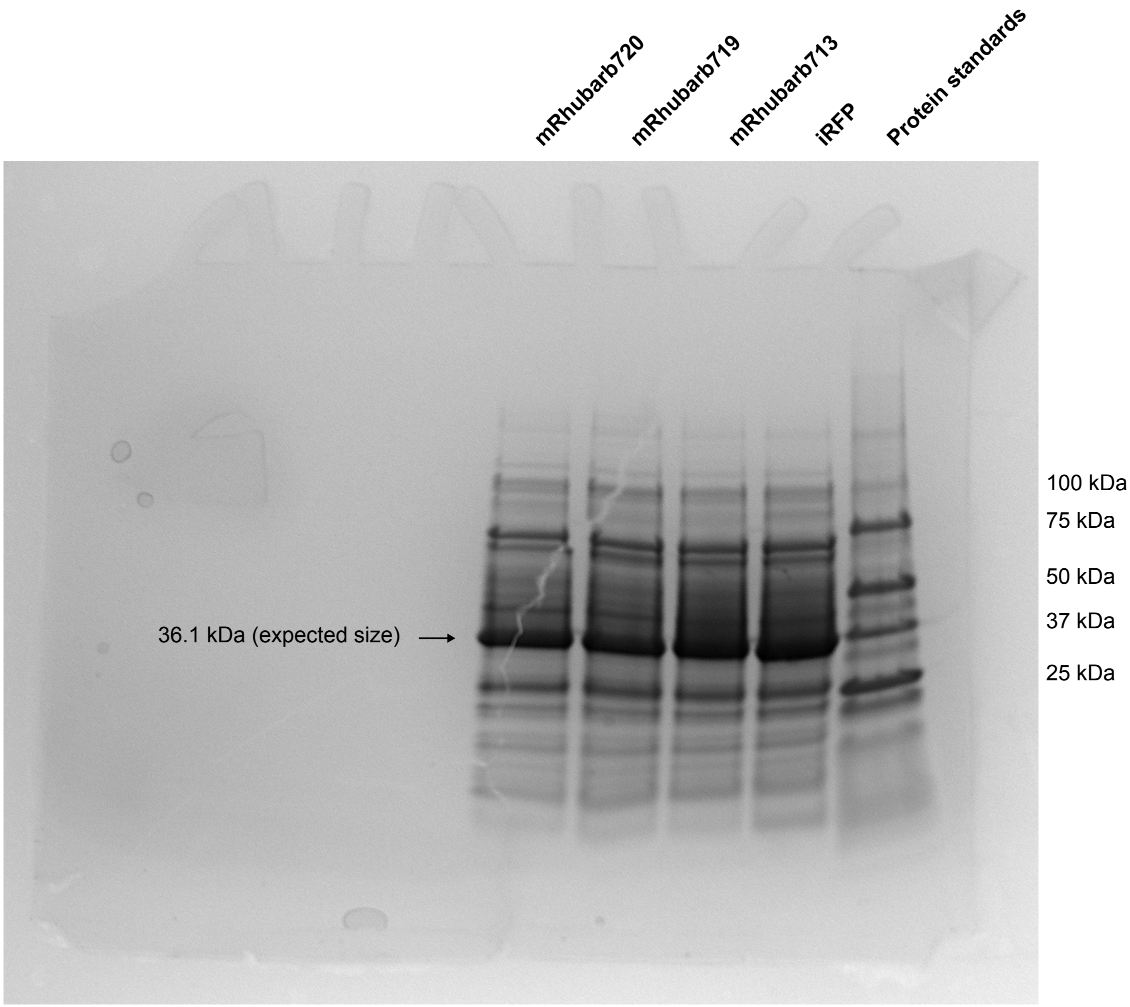

Supplementary Figure S3. Coomassie-stained SDS-PAGE of Ni-NTA purified iRFP variants expressed in *E. coli*. Each sample contains 15 µg total protein loaded. Densitometry analysis using ImageJ software on the major 36.1 kDa band in each sample was used to determine the relative concentration of each mutant relative to iRFP. This ensured that an equimolar amount of protein was loaded into all fluorescence assays.

BV interacting group	Spectral state	DrBphP	PaBphP	RpBphP2	iRFP
Ring B propionate oxygens	Pr	Y216	Y203	Y211	Y211
	Pfr	R222	R209	R217	R217
	Pr	R254	R241	R249	R249
	Pr	S257	S244	S252	S252
Ring C propionate oxygens	Pfr	Y176	Y163	Y171	Y171
	Pr	H260	H247	H255	H255
	Pr	S272	S259	T267	T267
	Pr	S274	S261	S269	S269
	-	I287	F274	I282	I282
	Pfr	A288	S275	A283	V283
Ring D Oxygen, Nitrogen	Pfr	H290	H277	H285	H285
	Pfr	H201	Q188	L196	L196
	Pfr	F203	Y190	F198	Y198
	Pfr	D207	D194	D202	T202
	-	I208	I195	I203	V203
	Pfr	Y263	Y250	Y258	F258
	Pr	R466	R457	R466	-
	Pfr	-	R453	R462	-
	Pfr	S468	S459	S468	-

Supplementary Table S1: Homologous residues from several BphPs and their interaction with BV groups in the Pr and Pfr states

iRFP-MAX-Y171F173	GAAGTCGGAGGCGNNGCGGNNGATCATCACCCGATCGAA
iRFP-MAX-K193	GTGCAGGCCTAGGNNTGACTCGACCTCGGCGCACCGAT
iRFP-MAX-Y198T202V203	CGGGCCTGCGCCGGGNNGNNTGAGGCAGGGNNGTGCAGGCCTAGTTTT
iRFP-MAX-R217	CGGGAATGATGNNTACCGGGTTGATGGTATA
iRFP-MAX-T267S269	CGCCGCGCAAAATCGAGATGNNCATGNNGCCGTGCATGCCTATGTT
iRFP-MAX-R249	GACGGGCGAGACGCTGNNCAGGATGGCGAAGCTA
iRFP-MAX-L281V283H285	CGGCGTTCGGTGGNNGCAGNNGATGNNTCCCCACAGTCGCTCGCCGCGCAAAAT
iRFP-MAX-V6A7R8Q9P10	GGTCAAGAGGTCGNNGNNGNNGNNGNNGGATCCTTCAGCCAT
iRFP-MAX-R172A174S175D176F177S178E180	CTCTGCGATCACGNNGCCGNNGNNGNNGNNGNNGAAGNNATAGATCATCACCCGATGCAA
V6-A7-R8-Q9-P10	GGCTCATCGTCGCAGGTCAAGAGGTCGNNGNNGNNGNNGNNGGATCCTTCAGCCATCGGATCCTGGC
Y171-R172-F173-A174-S175-D176-F177-S178-E180	CGGCGCACCGATCCTCTGCGATCACGNNGCCGNNGNNGNNGNNGNNGNNGNNGNNGATCATCACCCGATCGAAGCCGGTAATCTTC
K193-Y198-T202-V203	GACGGGCCTGCGCCGGGNNGNNTGAGGCAGGGNNGTGCAGGCCTAGGNNTGACTCGACCT
T267-S269-L281-V283-H285	CGACGTAGTACGGCGTTCGGTGGNNGCAGNNGATGNNTCCCCACAGTCGCTCGCCGCGCAAAATCGAGATGNNCATGNNGCCGTGCATGCCTATGTT
R217	CGGCCGATAATTGATATCGGGAATGATMNNTACCGGGTTGATGGTATAGAGCC
R249	TCCAGATGGACGGGCGAGACGCTMNNCAGGATGGCGAAGCTAAGATCAA
Y171A	AAGTCGGAGGCGAAGCGAGCGATCATCACCCGATCGAAG
L196Q-T202D-V203I-Y211A	GGGTTGATGGTAGCGAGCCGACGGGCCTGCGCCGGAATATCTGAGGCAGGATAGTGCTGGCCTAGTTTTG
BP22-R249A-H255A-F258Y-T267A-S269A	GCAAAATCGAGATCGCCATCGCGCCGTGCATGCCTATGTTGCGCATGTATTCCAGAGCGACGGGCGAGACGCTAGCCAGGATGGCG
BP23-I282F-V283S	CGTTCGGTGATGGCAAGAGAACAATCCCCACAGTCGCTC
BP24-L196Q-T202D-Y211A	GGGTTGATGGTAGCGAGCCGACGGGCCTGCGCCGGCACATCTGAGGCAGGATAGTGCTGGCCTAGTTTTG
BP25-V283S	CGTTCGGTGATGGCAAGAGATCAATCCCCACAGTCGCTC
BP26-Y171Y	AAGTCGGAGGCGAAGCGATAGATCATCACCCGATCGAAG
BP27-L196LQ-T202D-V203VI-Y211A	GGGTTGATGGTAGCGAGCCGACGGGCCTGCGCCGGAAYATCTGAGGCAGGATAGTGCWGGCCTAGTTTTG
BP28-L196LQ-T202D-V203VI-Y211Y	GGGTTGATGGTATAGAGCCGACGGGCCTGCGCCGGAAYATCTGAGGCAGGATAGTGCWGGCCTAGTTTTG
BP29-R249R-H255H-F258FY-T267TA-S269SA	GCAAAATCGAGATCGMCATCGYGCCGTGCATGCCTATGTTGCGCATGWATTCCAGATGGACGGGCGAGACGCTGCGCAGGATGGCG
BP30-R249A-H255H-F258FY-T267TA-S269SA	GCAAAATCGAGATCGMCATCGYGCCGTGCATGCCTATGTTGCGCATGWATTCCAGATGGACGGGCGAGACGCTAGCCAGGATGGCG
BP31-R249R-H255A-F258FY-T267TA-S269SA	GCAAAATCGAGATCGMCATCGYGCCGTGCATGCCTATGTTGCGCATGWATTCCAGAGCGACGGGCGAGACGCTGCGCAGGATGGCG
BP32-R249A-H255A-F258FY-T267TA-S269SA	GCAAAATCGAGATCGMCATCGYGCCGTGCATGCCTATGTTGCGCATGWATTCCAGAGCGACGGGCGAGACGCTAGCCAGGATGGCG
BP33-I282IF-V283V	CGTTCGGTGATGGCAAACGAWCAATCCCCACAGTCGCTC
BP34-I282IF-V283S	CGTTCGGTGATGGCAAGAGAWCAATCCCCACAGTCGCTC

Supplementary Table S2. List of PFunkel mutagenesis primers
